# Noninvasive genotyping and early disease dynamics demonstrate the efficacy of ibrutinib in combination with immunochemotherapy in patients with mantle cell lymphoma treated in the TRIANGLE trial

**DOI:** 10.1038/s41375-025-02787-0

**Published:** 2025-11-03

**Authors:** Mouhamad Khouja, Elisa Genuardi, Simone Ferrero, Anna Laqua, Beatrice Alessandria, Onno J. H. M. Verhagen, Christa H. E. Homburg, Ramón García Sanz, Alejandro Medina Herrera, Vincent H. J. van der Velden, Maria Gomes da Silva, Paula Gameiro, Jeanette Doorduijn, Eva Giné, Carlo Visco, Monika Brüggemann, Claudia D. Baldus, Marco Ladetto, Christian Schmidt, Martin Dreyling, Linmiao Jiang, Eva Hoster, Nikos Darzentas, Karol Pal, Guranda Chitadze, James Peter Stewart, David Gonzalez, Christiane Pott

**Affiliations:** 1 Second Medical Department, University Hospital Schleswig-Holstein, Kiel, Germany; 2 Department of Molecular Biotechnologies and Health Sciences - Hematology Division, Università di Torino, Torino, Italy; 3 Department of Immunocytology, Sanquin Diagnostic Services, Amsterdam, The Netherlands; 4 CIBERONC, Hospital Universitario de Salamanca-IBSAL, Universidad de Salamanca, Salamanca, Spain; 5 Laboratory Medical Immunology, Department of Immunology, Erasmus MC, University Medical Center Rotterdam, Rotterdam, The Netherlands; 6 Haematology Unit, Instituto Português de Oncologia de Lisboa Francisco Gentil, Lisbon, Portugal; 7 Department of Hematology, Erasmus MC Cancer Institute, University Medical Center Rotterdam, Rotterdam, Netherlands; 8 Hematology Department, Hospital Clínic de Barcelona, IDIBAPS, Barcelona, Spain; 9 Department of Engineering for Innovative Medicine, University of Verona, Verona, Italy; 10 Clinical Research Unit CATCH ALL, Christian-Albrechts-University of Kiel, Kiel, Germany; 11 SC Ematologia Azienda Ospedaliera Santi Antonio e Biagio e Cesare Arrigo, Alessandria, Italy; 12 Department of Medicine III, University Hospital, Ludwig-Maximilian-University, Munich, Germany; 13 Institute for Medical Information Processing, Biometry, and Epidemiology (IBE), LMU University Munich, Munich, Germany; 14 CEITEC MU - Central European Institute of Technology, Masaryk University, Brno, Czech Republic; 15 Precision Medicine Centre, Patrick G Johnston Centre for Cancer Research, Queen’s University Belfast, Belfast, United Kingdom

**Keywords:** Cancer genomics, Translational research, Risk factors, Genetics research

## Abstract

Adding ibrutinib to first-line immunochemotherapy (Ibru-R-chemo) showed superiority in younger mantle cell lymphoma (MCL) patients in the TRIANGLE trial (NCT02858258). To investigate response mechanisms and kinetics across treatment arms, we genotyped 57 patients from cell-free (cf)DNA using targeted-capture sequencing and investigated measurable residual disease (MRD) in cfDNA and peripheral blood by targeted-sequencing and qPCR. Pre-treatment cfDNA and circulating tumor (ct)DNA levels predicted outcomes, and precisely genotyped all patients. Circulating tumor cell (CTC)-clearance was more frequent and rapid than ctDNA-clearance across arms. At interim staging (IS), 55% of patients were ctDNA-positive while 35% and 41% were CTC-positive by qPCR and immunoglobulin gene (IG)-NGS. At end of induction, 43% were ctDNA-positive, while 15% (qPCR) and 25% (IG-NGS) were CTC-positive. MRD by qPCR was most predictive for outcomes. Ibru-R-chemo seemed to overcome *TP53*^*mut*^*-*mediated risk (hazard ratio 1.9 vs. 10) and induce early MRD response, represented by enhanced CTC (71% vs. 57%) and ctDNA clearance (59% vs. 24%) at IS. Flow-cytometry-based immunomonitoring showed ibrutinib’s influence on inhibitory T-cell phenotypes, showing ≥25% reduction in PD1+ and PD1+ KLRG1+ CD4+-T-cells in four patients. Taken together, besides direct anti-B-cell efficacy, ibrutinib improves chemotherapy efficiency by reconstituting an effective immune system and enhancing immune cell control.

## Introduction

Mantle cell lymphoma (MCL) is a rare non-Hodgkin’s lymphoma (NHL) subtype characterized by rapid growth of abnormal B-cells in the mantle zone of the lymph node (LN), mainly driven by cyclin D1 overexpression upon the *IGH::CCND1* translocation [1, 2]. High-risk features like high MCL international prognostic index (MIPI), blastoid morphology, high Ki-67 index, and *TP53* alterations are the most adverse predictive factors, even after cytarabine dose-intensification or autologous stem-cell transplantation (ASCT) [3–5].

The TRIANGLE trial (NCT02858258) of the European (E)MCL network showed significant and clinical superiority of Bruton’s Tyrosine kinase inhibitor (BTKi) ibrutinib addition to immunochemotherapy with ASCT (arm A + I) or without (arm I), compared to immunochemotherapy+ASCT (arm A). This supports ibrutinib as standard-of-care during induction and maintenance in first-line treatment of younger MCL patients [6, 7]. It remains unclear, how ibrutinib influences disease kinetics during induction with short-term exposure (days 1–19 of the three R-CHOP cycles of R-CHOP/R-DHAP) and what impact it has on cellular dynamics and measurable residual disease (MRD) response.

Besides high-risk biological markers, MRD detection by allele-specific quantitative (q)PCR allows sensitive longitudinal monitoring of tumor load and kinetics. In prospective clinical trials, MRD evolved as one of the strongest outcome-predictive parameters [8–10]. It serves as a dynamic indicator for induction efficacy, allowing risk-adapted treatment. The gold-standard for MRD in MCL is qPCR of patient-specific immunoglobulin gene (IG) rearrangement, assessing circulating lymphoma cells [11, 12].

Novel research highlights the feasibility of plasma cell-free (cf)DNA and circulating tumor (ct)DNA for biomarker identification and genetic assessment in classical Hodgkin’s (cHL) and B-NHL, like diffuse-large B-cell lymphoma (DLBCL) and MCL [13–18].

Here, we used cfDNA for pretreatment molecular profiling and MRD assessment under highly-efficient treatments including ibrutinib, to (a) gain insights into ibrutinib’s mechanism of action in MCL and (b) assess the clinical benefit of MRD assessment in cfDNA over standard MRD techniques in genomic (g)DNA derived from peripheral blood (PB) and bone marrow (BM) cells.

## Materials and methods

### Patient collection and study design

Fifty-seven advanced-stage MCL patients treated within the TRIANGLE trial were selected according to plasma availability, PB involvement ≥10% at diagnosis to allow comparative genotyping in tumor cells and plasma, and available MRD data in PB and BM determined by qPCR according to the study plan. Twenty-three patients were treated in arm A, while seventeen were treated in arms A + I and I, each. Samples were collected in EDTA or cfDNA stabilization tubes (cfDNA BCT, STRECK®) and stored in national reference laboratories for prospective MRD and molecular analyses. Analyzed samples were from Germany, Italy, Netherlands, Spain, and Portugal, all members of the European study group. Ethical approval was obtained from the ethics committees of participating centers.

### DNA extraction and quantification

Plasma was isolated from STRECK® tubes with centrifugation for 5 min, 2000 × *g* followed by 10 min, 16,000 × *g*, 4 °C, and stored at −80 °C. cfDNA was purified from ≤4 mL plasma with the QIAmp Circulating Nucleic Acid Kit (Qiagen, Germany) and quantified using multiplex ddPCR [19]. cfDNA quantity was reported as the number of haploid genome equivalents per milliliter of plasma (hGE/mL) and expressed as base-10 logarithm. cfDNA quality was assessed on an Agilent Bioanalyzer 2100 using the high-sensitivity DNA kit (Agilent Technologies, Germany). gDNA was extracted from an EDTA tube by standard methods.

### IGH marker identification and MRD assessment using the EuroClonality (EC-)IG-NGS assay

IGH-VJ rearrangements were assessed in cellular and cell-free compartments using the EC-IG-NGS assay [20, 21]. For screening, 100 ng gDNA or 3000 hGE cfDNA was used in one-step amplicon-based NGS approach using EC-IGH-VJ-FR1 or IGH-VJ-FR3cf primers, respectively [21]. For MRD assessment in PB, 8 µg gDNA was used in four replicates using the IGH-VJ-FR1 primers to achieve a sensitivity of 10^−6^. For longitudinal ctDNA analysis, 4990 hGE cfDNA was analyzed in median using the EC-IGH-VJ-FR3cf primer set (range: 1200-16174 hGE). Amplicon libraries were sequenced using 2 × 250 bp chemistry on Illumina MiSeq (Illumina, UK). A central in-tube quantification and quality control (cIT-QC) was used for quantification [22]. Data was analyzed using ARResT/Interrogate with adaptations for cfDNA [23]. Lymphoma-specific IGH clonotypes were selected when representing ≥5% annotated IG reads and ≥1% cells after normalization using cIT-QC. Samples were considered MRD positive by detecting ≥1 lymphoma-specific IGH reads.

### DNA library preparation and targeted-capture sequencing

DNA sequencing libraries were prepared from 100 ng diagnostic gDNA using KAPA HyperPlus Library Preparation Kit (Kapa Biosystems, Roche, Germany) [24]. DNA libraries from ≥3000 hGE cfDNA were prepared on an AVENIO Edge® using the AVENIO Edge HyperPrep Kit (Roche, Germany). gDNA and cfDNA libraries were subject to targeted-capture enrichment by hybridization using the EuroClonality-NDC assay (Univ8® genomics, UK, Supplementary Tables S1, S2) and sequenced using a 2 × 75 bp or 2 × 100 bp chemistry on Illumina NextSeq500 and NovaSeq6000 platforms (Illumina, UK) with a median depth of 773× (range 307–2080×) and 2657× (range 932–4660×), respectively. Data was processed using the EuroClonality-NDC ARResT/Interrogate pipeline [23]. An assay-specific panel-of-normals from gDNA and cfDNA was used to exclude sequencing artifacts. For molecular profiling, IG clonotypes were called when presenting ≥6 unique consensus reads and ≥4% of annotated IG reads, structural variants were called when presenting ≥4 unique reads and single nucleotide variants were called when presenting ≥3 unique reads, and a variant allele frequency (VAF) of ≥1% in cfDNA and ≥4% in gDNA.

For MRD at interim staging (IS) and end of induction (EoI), 10,275hGE cfDNA was used in median for library preparation (range 3050–15,000hGE) and sequenced with a median depth of 2832× (range 1585–3927×) after targeted enrichment using EuroClonality-NDC. The bioinformatics pipeline was optimized to enable higher sensitivity by calling mutations with ≥1 read and VAF ≥ 0.02% and a parallel tracing of junction sequences of IG rearrangements and/or *IGH::CCND1* fusions. Final ctDNA levels were reported as the number of mutated molecules per milliliter of plasma (MMPM) and expressed on base-10 logarithm.

### MRD assessment by qPCR

MRD analysis in PB and BM by allele-specific qPCR was performed according to the trial’s protocol in national reference labs by standardized methods (sensitivity 10^−4^–10^−5^) following EuroMRD guidelines with clear definitions of positivity in quantitative range (QR) and below (B)QR (www.euromrd.org) [25].

### Flow-cytometric immunophenotyping

T-cell composition was investigated using multi-parametric flow-cytometry by analyzing DMSO-conserved PB/BM cells obtained at diagnosis, IS and EoI. Briefly, cells were thawed, washed in PBS/0.09%NaN_3_/0.2%BSA, and incubated for 30 min, RT, with antibody cocktail containing BD Horizon Brilliant Stain Buffer Plus (BD Biosciences, USA) and monoclonal antibodies directed against CD3 (UCHT1), CD4 (REA623), CD8 (SK1), CD14 (63D3), CD16 (3G8), CD19 (SJ25C1), CD25 (M-A251), CD27 (O323), CD28 (CD28.2), CD38 (HB-7), CD39 (TU66), CD45 (HI30), CD45RA (HI100), CD56 (5.1H11), CD57 (QA17A04), CD95 (DX2), CD127 (A019D5), CCR7 (G043H7), HLA-DR (L243), PD-1 (EH12.2H7), KLRG1 (13F12F2), TIM-3 (F38-2E2), and TCRγδ (REA591). Followed by 10 min incubation at RT with BD FACS Lysing Solution (BD Biosciences, USA), and wash. Samples were acquired and unmixed on a Cytek® Northern Lights™ spectral flow cytometer using SpectroFlo v3.1.0 software. Unmixed data was analyzed using Infinicyt™ v2.0.5.d for identification and export of T-cells, afterwards, FlowJo v10.8 was used for additional quality control using PeacoQC v1.4.1 plugin and downstream analysis of T-cell subpopulations.

### Statistical analyses

Failure-free survival (FFS), the primary outcome in TRIANGLE, was defined as time from randomization to stable disease, progression, or death from any cause, whichever occurs first. Overall survival (OS) was defined by time from randomization to death from any cause. FFS and OS were estimated with Kaplan–Meier estimates and compared using Log-rank tests. To account for potential confounding, multivariable Cox proportional hazards regression models were performed with adjustment for MIPI score, rituximab maintenance and p53 status. Patients from ibrutinib-containing arms (arm A + I/I) were combined for assessment of statistical significance since the analysis consisted of samples pre-ASCT and due to the comparable outcomes of the two experimental arms. Statistical significance between groups for cfDNA and ctDNA levels was estimated using Mann–Whitney U tests for unpaired samples. No adjustment for multiple testing was applied in this exploratory study, as each hypothesis addressed an independent research question with distinct biological or clinical relevance, and a higher statistical power was maintained. All results were reported transparently irrespective of statistical significance of tests performed [26]. Optimal pretreatment cf-/ctDNA level cut-off was determined by repeatedly (2000 times) sampling patients with replacement and, in each iteration, selecting the threshold that best separated patients based on FFS using the log-rank test. Data analysis and presentation were performed using R version 4.4.1 (www.R-project.org) and GraphPad Prism 8 (www.graphpad.com).

## Results

### Patient characteristics

Fifty-seven of 870 patients enrolled in the TRIANGLE trial were selected for this molecular analysis based on PB involvement ≥10% at diagnosis and plasma availability for IS and EoI. Compared to the overall study population, selected patients had significantly higher WBC and LDH levels but comparable FFS and OS outcomes (median survival not reached) (Table 1, Supplementary Fig. S1A, B). Similar to the results from the overall study population [6]. selected patients in arm A (*n* = 23) had inferior FFS and OS compared to selected patients in arms A + I (*n* = 17) and I (*n* = 17) (HR:1.82 (FFS) and 2.53 (OS)) (Supplementary Fig. S1C, D).

**Table 1:** Baseline patient characteristics.

Variable	Value	Patients with cfDNA analysis (*N* = 57)		Patients without cfDNA analysis (*N* = 813)		*P* value
Induction	Arm A (*n*, %)	23	40%	265	33%	0.51
	Arm A + I (*n*, %)	17	30%	275	34%	
	Arm I (*n*, %)	17	30%	273	34%	
Rituximab Maintenance		39	68%	464	57%	0.098
Study groups	GLSG	24	42%	262	32%	0.00016
	PLRG	2	4%	36	4%	
	CLSG	0	0%	16	2%	
	FIL	10	18%	196	24%	
	HOVON	12	21%	87	11%	
	Nordic lymphoma group	0	0%	110	14%	
	SAKK	0	0%	32	4%	
	GELTAMO	7	12%	66	8%	
	Israeli lymphoma group	0	0%	6	1%	
	Centers without study group	2	4%	2	0%	
Age (years)	Median, Min-Max	56	31–65	57	27–68	0.55
Sex	Male (*n*, %)	46	81%	616	76%	0.52
Stage	II (*n*, %)	0	0	41 (*n* = 808)	5%	0.03
	III (*n*, %)	0	0	74 (*n* = 808)	9%	
	IV (*n*, %)	56 (*n* = 56)	100%	693 (*n* = 808)	86%	
B-symptoms	Present (*n*, %)	16	28%	221 (*n* = 803)	28%	>0.99
ECOG	0 (*n*, %)	35	61%	599	74%	0.077
	1 (*n*, %)	22	39%	201	25%	
	2 (*n*, %)	0	0%	12	1%	
LDH (ULN)	Median, Min-Max	1.08	0.65–2.92	0.91	0.36–8.46	<0.0001
WBC (G/L)	Median, Min-Max	12.1	3.7–242.6	7.17	0.16–599	0.0001
Ki-67	Median, Min-Max	20 (*n* = 52)	1–95	18 (*n* = 718)	0–98	0.55
Ki-67	≥30%	20 (*n* = 52)	38%	224 (*n* = 718)	31%	0.28
Cytology	Blastoid (including pleomorphic)	8 (*n* = 52)	15%	85 (*n *= 727)	12%	0.38
MIPI score	Median, Min-Max	5.88	4.51–7.18	5.6	4.25–8.1	<0.0001
MIPI	Low (*n*, %)	22	39%	482	59%	0.00086
	Intermediate (*n*, %)	17	30%	219	27%	
	High (*n*, %)	18	32%	112	14%	
P53 expression	> 50% (*n*, %)	6 (*n* = 41)	15%	71 (*n* = 506)	14%	0.82

*GLSG* German Lymphoma Study Group, *PLRG* Polish Lymphoma Research Group, *CLSG* Czech Lymphoma Study Group, *FIL* Fonazione Italiana Linfomi, *HOVON* Stichting Hemato-Oncologie voor Volwassenen Nederland, *SAKK* Swiss Cancer Institute, *GELTAMO* Soanish Lymphoma Group.

### ctDNA provides a reliable source for genotyping of MCL

ctDNA assessment by targeted-capture sequencing allowed the identification of *IGH*::*CCND1* fusion in 56/57 patients (Supplementary Table S3). One patient with undetectable *IGH*::*CCND1* fusion had an unknown translocation status by fluorescence in-situ hybridization in the available BM sample but showed clonal IGH-VJ and IGH-DJ rearrangements. Clonal IGH-VDJ rearrangements were detected in plasma in all 57 (100%) patients, IGH-DJ in 1/57 (2%), IGK in 57/57 (100%) and IGL in 33/57 (58%) patients (Supplementary Table S4). IGH-VJ rearrangements were confirmed by the (EC-)IG-NGS assay in both, CTC and ctDNA.

**Fig. 1:**
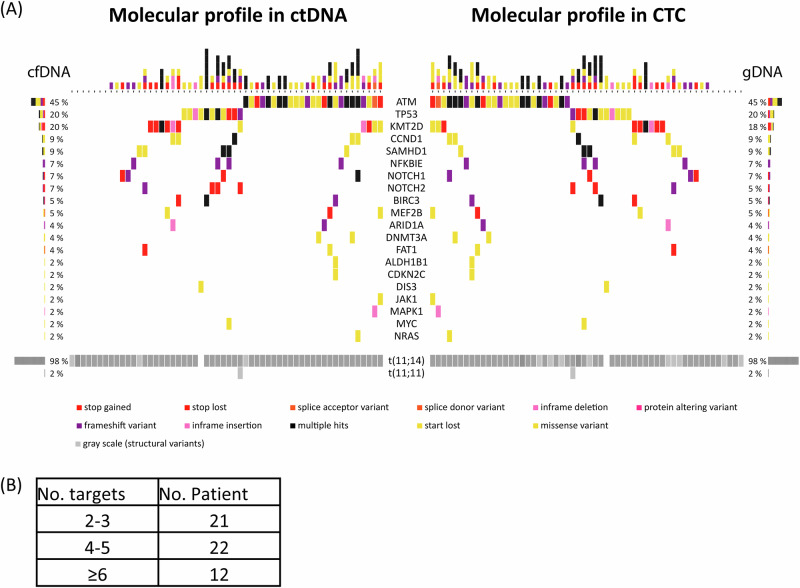
Targeted sequencing of 57 MCL patients from ctDNA and confirmed in CTC using the EuroClonality NDC approach. **A** Heatmap with the mutation pattern of the top 20 recurrently mutated genes in addition to the structural variants. Horizontal bar graph shows the frequency of somatic mutations for each gene in both compartments. **B** Number of identified MRD markers per patient.

Somatic variants were identified in cfDNA in 54/57 (95%) patients, comprising mutations in MCL target genes such as *ATM* (47%), *TP53* (19%), *KMT2D* (21%), *CCND1* (10%), *SAMHD1* (8%) and 18 others (Fig. 1A, Supplementary Table S5), while only three patients had no detectable variants in target genes.

Identified somatic variants were confirmed in the parallel analyzed gDNA from CTC in 51/57 patients. Remarkably, 11 somatic variants were only detected in ctDNA of six patients with a median VAF of 11.15% and comprised mainly MCL target genes such as *TP53*, *NOTCH2*, and *ATM* (Supplementary Fig. S2).

Genomic profiles of four patients were cross-validated by analyzing DNA from diagnostic LN FFPE samples. Identified variants in FFPE DNA confirmed mutation profiles of ctDNA and CTC in 3/4 patients. In one patient, FFPE DNA showed three additional variants in *BIRC3*, *KLF2*, and *SF3B1*, all three were also detected in the corresponding cfDNA with an average VAF of 0.5%, below the pre-defined threshold for variant detection (Supplementary Table S6) but not in CTC. In median, six MRD markers were identified per patient (range: 3–10) (Fig. 1B).

### Clinical impact of pretreatment ctDNA

**Fig. 2:**
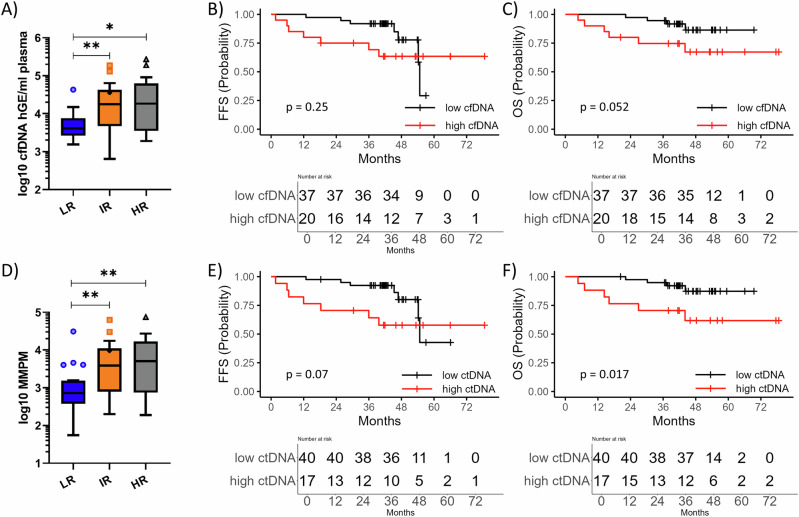
Clinical impact of ctDNA analysis. Association of pretreatment cfDNA (**A**) and ctDNA (**D**) levels with MIPI risk groups. MMPM mutated molecules per mL plasma, LR low-risk, IR intermediate risk, HR high-risk, asterisks indicate to significance level. Kaplan–Meier estimates of FFS and OS rates depicted by pretreatment cfDNA levels over 4.19 log_10_hGE/mL (**B**, **C**) or ctDNA levels over 3.72 log_10_MMPM (**E**, **F**). High cfDNA and ctDNA levels were represented by a red line, while low levels were represented by a black line.

Baseline cfDNA levels correlated with MIPI risk groups (*r* = 0.35, *p* = 0.007) and were in median 3.61 log_10_hGE/mL for low-risk, 4.24 log_10_hGE/mL for intermediate risk and 4.26 log_10_hGE/mL for high-risk (Fig. 2A). cfDNA^high^ (>4.19 log_10_hGE/mL) significantly predicted OS rates (*p* = 0.043, HR:3.42[0.96–12.15]) and showed a trend towards inferior FFS (*p* = 0.25, HR:1.82[0.65–5.05]) (Fig. 2B, C).

Similarly, ctDNA levels correlated with MIPI risk groups (*r* = 0.4, *p* = 0.002) and were in median 2.85 log_10_MMPM for low-risk, 3.58 log_10_MMPM for intermediate risk and 3.66 log_10_MMPM for high-risk (Fig. 2D). Importantly, ctDNA^high^ (>3.72 log_10_MMPM) significantly predicted inferior OS (*p* = 0.0134, HR:4.33[1.22–15.37], 3-years OS: 70% vs. 95%) and showed a trend towards impaired FFS (*p* = 0.07, HR:2.42[0.87–6.72], Fig. 2E, F). By analyzing patients according to treatment arm, the impact of ctDNA^high^ and cfDNA^high^ was associated with inferior OS in patients treated in arm A (*p* = 0.035, both) but not in arms A + I/I (*p* = 0.42 (cfDNA^high^) and 0.21 (ctDNA^high^)).

In a multivariable cox regression model, adjusting for MIPI and p53 status, ctDNA levels predicted inferior FFS and OS rates (ctDNA^high^: FFS: *p* = 0.018, HR:6.17[1.36–28.09], OS: *p* = 0.056, HR:5.46[0.95–31.32]).

### Longitudinal ctDNA assessment and clinical prognostic value

**Fig. 3:**
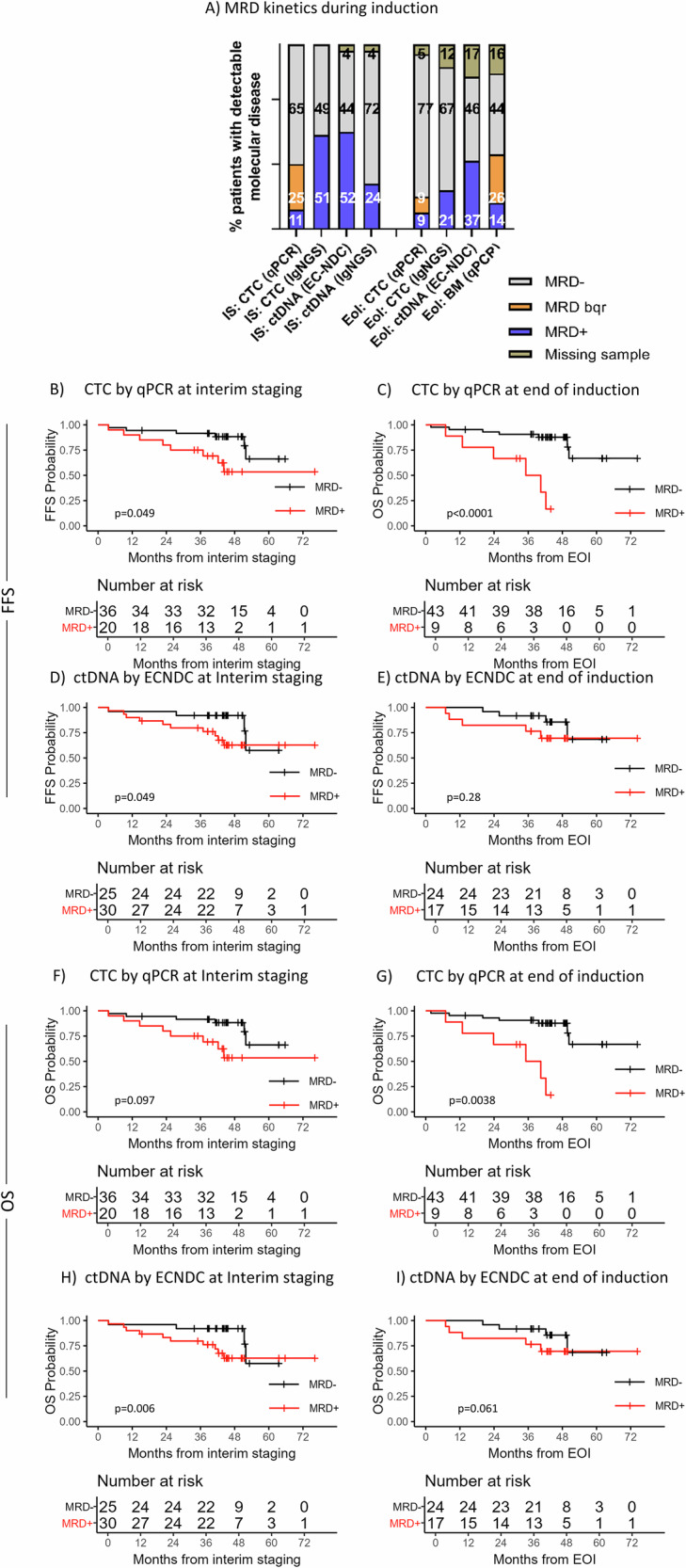
Clinical impact of MRD assessment in ctDNA and CTC. **A** Levels of detectable MRD by EuroClonality-NDC for ctDNA and qPCR and IG-NGS for CTC in two induction timepoints. IS interim staging, EoI end of induction. Kaplan–Meier estimates demonstrating the prognostic value of MRD assessment at interim staging and end of induction for adverse FFS rates by qPCR (**B**, **C**) and ctDNA (**D**, **E**) as well as the prognostic value of MRD assessment for adverse OS rates by qPCR (**F**, **G**) and ctDNA (**H**, **I**) in the analyzed TRIANGLE subcohort. MRD positivity was represented by a red line, while patients with undetectable MRD were represented by a black line.

At IS, ctDNA was detected by EuroClonality-NDC in 30/55 (55%) and by EC-IG-NGS in 14/55 (26%) patients. CTC was detected by qPCR in 20/57 (35%) and by EC-IG-NGS in 29/57 (51%) patients (6/29 had MRD between 10^−5^–10^−6^) (Fig. 3A).

At EoI, ctDNA was detected by EuroClonality-NDC in 20/47 (43%), while CTC was detected by qPCR in 8/53 (15%) and by IG-NGS in 13/53 (25%) patients (6/13 had MRD between 10^−5^–10^−6^). MRD in BM was detected by qPCR in 23/48 (48%) patients; 8/48 (17%) patients had quantifiable MRD and 15/48 (31%) patients were BQR (Fig. 3A). In summary, MRD was more frequently detected in plasma and BM compared to PB at both timepoints, even when EC-IG-NGS (sensitivity: 10^−06^) was applied.

Inferior FFS was significantly associated with CTC detection by qPCR at IS (*p* = 0.01, HR:3.47[1.17–10.30], 3-years FFS:79% vs. 89%) and EoI (*p* < 0.0001, HR:8.26[2.48–27.56], 3-years FFS:60% vs. 90%, Fig. 3B, C). ctDNA detection by EuroClonality-NDC showed a trend towards inferior FFS at IS (*p* = 0.11, HR:2.52[0.79–8.06]) and EoI (*p* = 0.28, HR:2.04[0.55–7.63]) (Supplementary Table S6, Fig. 3D, E).

OS was significantly associated with ctDNA-positivity by EuroClonality-NDC at IS (*p* = 0.026, HR:7.41[0.93–59.32]) and EoI (*p* = 0.029, HR:7.61[0.89–65.21]) and CTC detection by qPCR at IS (*p* = 0.03, HR:3.95[0.99–15.84]) and more pronounced at EoI (*p *= 0.0005, HR:7.33[1.94–27.72], Fig. 3F, I).

In multivariable Cox regression model adjusting for MIPI scores, rituximab maintenance (*n* = 39) and p53 expression status, only CTC-positivity by qPCR at EoI remained predictive for adverse FFS (*p* = 0.0089, HR:8.15[1.73–45]) and OS rates (*p* = 0.034, HR:7.09[1.16–43.23]) (Supplementary Table S6).

When analyzing treatment arms separately, despite none of the analyses in patients in arm A being significantly predictive, ctDNA-positivity by EuroClonality-NDC at IS and EoI showed higher risk of adverse FFS and OS than CTC detection (FFS: IS:2.57 vs. 1.9, EoI:4.13 vs. 4.12, OS: IS:Inf vs. 1.76, EoI:4.1 vs. 3.49), even after adjusting for MIPI scores and rituximab maintenance (*n* = 15/23, Supplementary Table S7). In ibrutinib-containing arms A + I/I, CTC-positivity by qPCR at IS (*n* = 10/34) and EoI (*n* = 4/31) was significantly predictive for adverse FFS and OS (IS: FFS: *p* = 0.018, HR:6.05[1.1–33.22], OS: *p* = 0.03, HR:7.68[0.8–37.8], EoI: FFS: *p* < 0.00001, HR:17.36[2.8–107], OS: *p* = 0.0004, HR:23.16[1.97–272], Table S7).

### Ibrutinib impacts disease kinetics and prognostic value of MRD response and *TP53* mutations

**Fig. 4:**
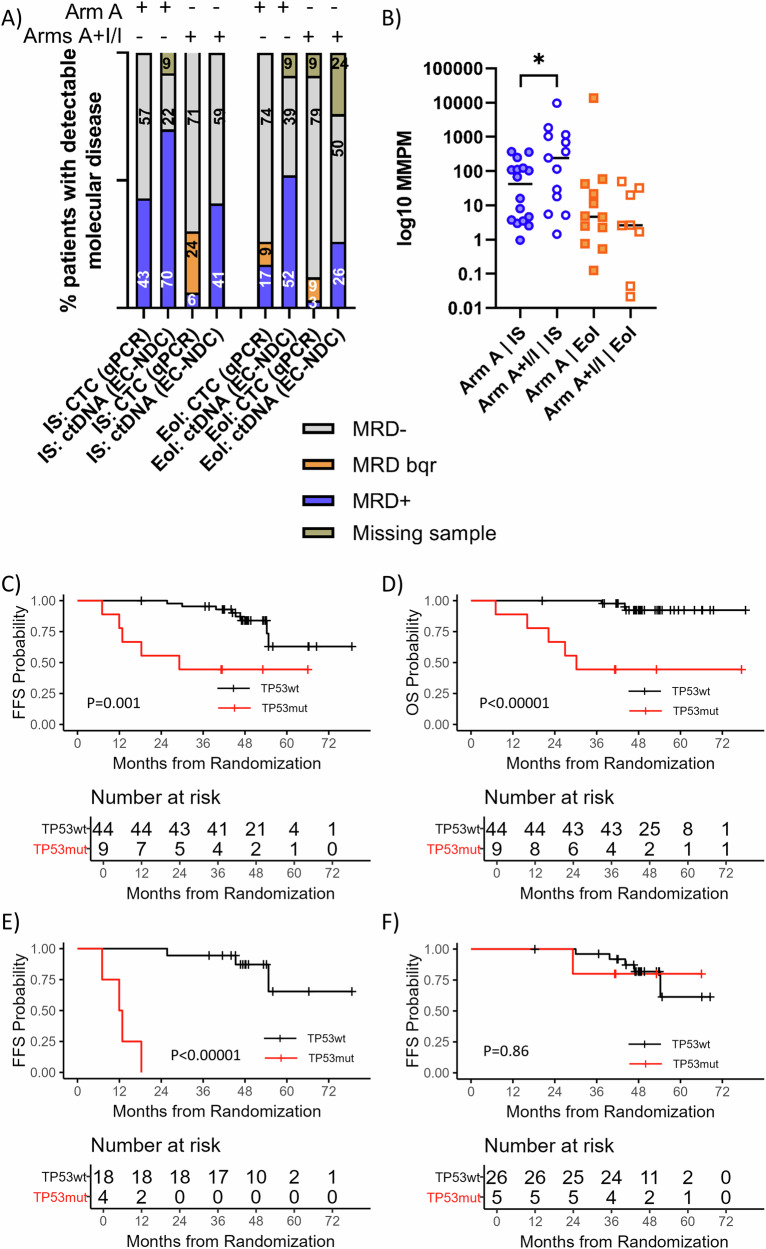
Ibrutinib’s impact on disease kinetics and the prognostic value of TP53 mutations. **A** Levels of detectable molecular disease assessed by qPCR for peripheral blood and EuroClonality-NDC for cfDNA depicted by treatment arm in two induction timepoints. IS interim staging, EoI end of induction. **B** ctDNA levels during induction timepoints, depicted by treatment arm; Blue dots represent interim staging and orange dots represent end of induction. Kaplan–Meier estimates of FFS (**C**) and OS (**D**) rates depicted by TP53 mutations the complete cohort and FFS rates depicted by TP53 mutations detected in arm A (**E**) and A + I/I (**F**). Aberrant cases were represented by a red line, while patients with wildtype genotypes were represented by a black line.

Investigating MRD dynamics by treatment arm revealed that ibrutinib-receiving patients (arms A + I/I; *n* = 34) showed faster and more effective clearance of CTC and ctDNA at IS and EoI than those receiving immunochemotherapy only (arm A, *n *= 23) (IS:71% vs. 57% (PB) and 59% vs. 24% (plasma), EoI:87% vs. 74% (PB) and 70% vs. 43% (plasma)) (Fig. 4A). At all conditions, ctDNA was more frequently detected than CTC.

Despite fewer ctDNA-positive patients at IS in arms A + I/I (*n* = 14/34) compared to arm A (*n* = 16/21), median ctDNA levels in ctDNA-positive patients were significantly higher in ibrutinib-treated patients (2.38 vs. 1.6 log_10_MMPM, *p* = 0.05, Fig. 4B). This might reflect the more rapid and effective response by ibrutinib in combination with immunochemotherapy in the LN compartment, taking place early during treatment, as ctDNA levels at EoI were comparable across treatment arms.

Identification of *TP53* mutations in ctDNA (11/57 patients) significantly predicted impaired FFS (*p* = 0.0018, HR:4.54[1.6–12.89], 3-years FFS: 45% vs. 93%) and OS (*p* < 0.0001, HR:10.08[2.78–36.49], 3-years OS: 45% vs. 98%) (Fig. 4C, D). However, this negative effect was only observed for patients in arm A (*n* = 5/23) (FFS: *p* < 0.0001, 18-months FFS: 20% vs. 100%, OS: *p* = 0.0005, 18-months OS: 40% vs. 100%) (Fig. 4E), but not in ibrutinib-treated patients (arms A + I/I, *n* = 6/34) (3-years FFS: 83% vs. 93%) (Fig. 4F, Supplementary Table S8).

### Ibrutinib might facilitate the reconstitution of a fit, competent immune system

To obtain indications of a potential positive influence of ibrutinib on T-cell function, we analyzed T-cell phenotypes during induction immunochemotherapy in eight patients (four receiving ibrutinib and four not) using spectral flow cytometry. BM was analyzed in two patients at diagnosis with no available PB cells. Since retrospective analysis of cryopreserved cells inherently limits the accuracy of absolute cell count measurements, we focused on the relative proportions of cell subsets.

**Fig. 5:**
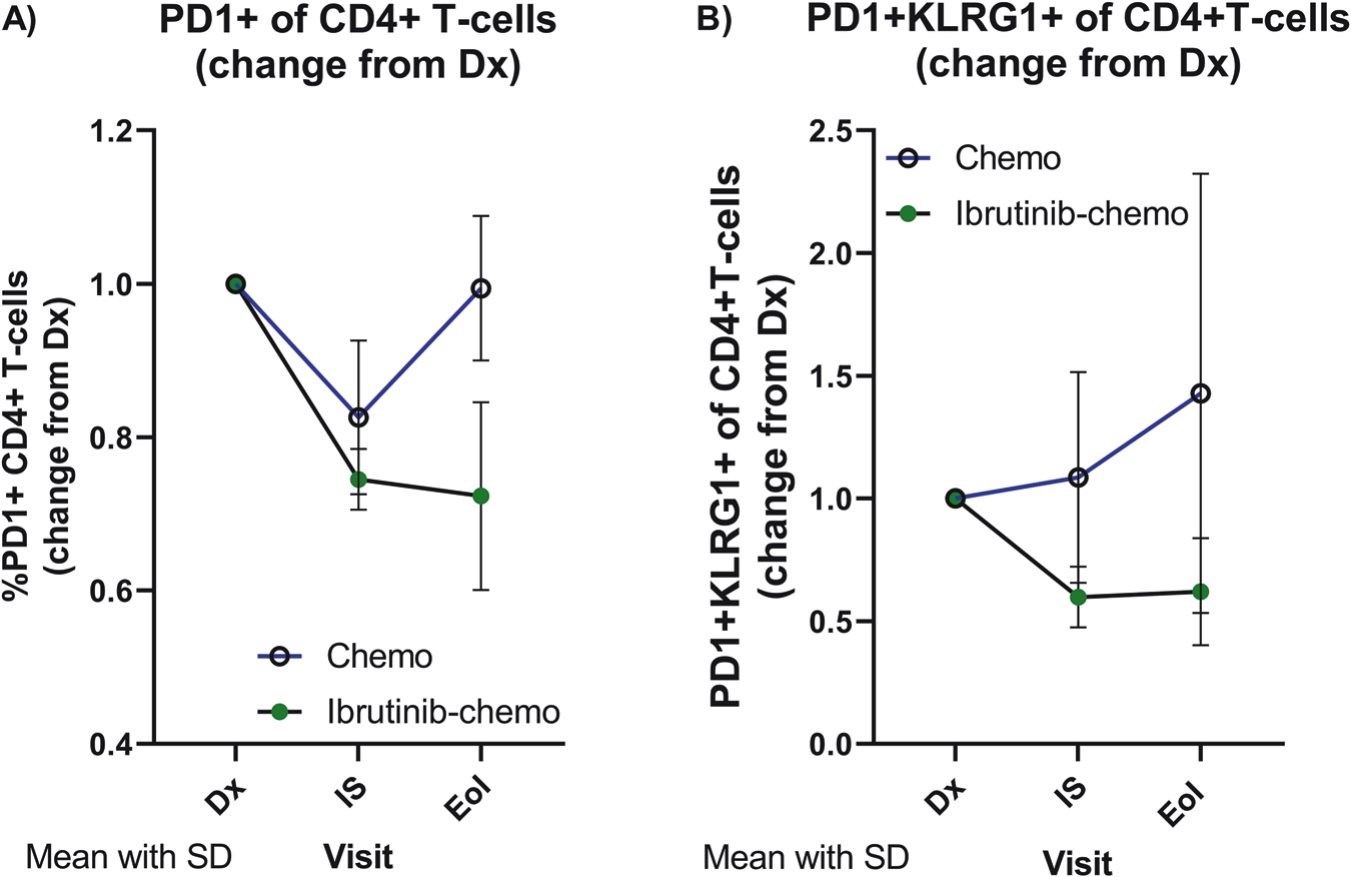
T-cell characterization during ibrutinib administration. Logarithmic presentation of **A** fold-changes of percentages of PD1 positivity in CD4+ cells among all CD3+ T-cells during treatment. **B** Fold-changes of percentages of PD1+/KLRG1+ CD4+ T-cells during treatment.

Among all CD3+ T-cells, the proportion of CD4+ T-cells expressing PD-1, an inhibitory checkpoint commonly associated with immune suppressive T-cell populations, decreased by 25% at IS and 28% at EoI in patients receiving ibrutinib, while persisting in patients receiving immunochemotherapy only (18% reduction at IS and only 1% at EoI) (Fig. 5A). Moreover, the proportion of PD1+ CD4+ T-cells co-expressing KLRG1, another co-inhibitory receptor together with PD1 marking more advanced stages of T-cell dysfunction [27], increased under immunochemotherapy but decreased under ibrutinib administration (IS: 1.08-fold vs. 0.59-fold, EoI: 1.42-fold vs. 0.62-fold) (Fig. 5B). Despite the small number of patients, our results suggest supportive effects of ibrutinib in restoring T-cell functionality.

## Discussion

The TRIANGLE trial demonstrated superiority of ibrutinib addition on outcomes of younger MCL patients in first-line settings [6, 7, 28, 29]. It remains unclear, how ibrutinib influences disease kinetics during induction with short-term exposure and which impact it has on MRD dynamics and response.

MRD in PB/BM is an early relapse predictor, providing a basis for risk stratification and interventional MRD-driven treatment in MCL [10, 30, 31]. ctDNA may add information about disease dynamics in MCL as demonstrated in cHL and DLBCL [13–15, 17, 32], particularly in highly effective treatments with profound depletion of circulating MCL cells.

Here, we investigated ctDNA for comprehensive genotyping and MRD assessment in a subset of MCL patients treated within the TRIANGLE trial.

Although MCL is not highly proliferative in most cases, pretreatment cfDNA levels were higher than baseline cfDNA levels we measured in patients with DLBCL [33]. This might in part be due a selection bias for patients. with ≥10% of PB infiltration, however more likely reflects the high tumor load of advanced MCL, as we demonstrated a significant correlation with MIPI score and a prognostic value of baseline cfDNA and ctDNA levels for outcomes. Importantly, baseline ctDNA levels predicted adverse outcomes in a multivariate analysis independently from MIPI scores and p53 expression status.

We demonstrated cfDNA’s feasibility for genotyping and biomarker identification in MCL, and that it might even better reflect the heterogeneous genotype of the disease, as shown in one representative case with mutations found in LN DNA and cfDNA but not in CTC and in six other cases with 11 additional variants in cfDNA. Thus, ctDNA offers an accessible tool for genotyping independent of LN tissue availability. The EuroClonality-NDC assay also enabled MRD marker screening from plasma, providing complete information on clonal IG rearrangements and genetic variants.

Plasma-based genotyping reliably detected *TP53* alterations, a validated adverse prognostic factor [5, 34, 35]. Interestingly, *TP53-*mediated adverse prognosis seemed abrogated in the ibrutinib-treated cohort, suggesting BTKi can partially overcome *TP53*-mediated treatment resistance. This aligns with findings in the whole TRIANGLE cohort, where patients with p53^high^ expression in arms A + I/I had superior FFS compared to patients in arm A [6]. Overcoming *TP53-*mediated adverse outcomes by BTKi was also recently reported by Ruan et al. where 5/6 patients with *TP53*^*mut*^ achieved complete response after 12-cycles of acalabrutinib-lenalidomide-rituximab [36]. BTKi’s pronounced effect could be related to NF-kB and PI3K-AKT pathways inhibition, reducing survival proteins levels (e.g. BCL-2 and BCL-XL) and inducing FOXO3a/Bim-mediated apoptosis. However, our findings warrant validation in the complete cohort due to the small sample size.

Adding ibrutinib to platin-based immunochemotherapy improved outcomes in the TRIANGLE trial. To understand disease dynamics and ibrutinib’s influence on tumor cell killing, we assessed MRD in ctDNA and CTC in parallel. The highly favorable outcome of ibrutinib-receiving patients can be partly attributed to enhanced disease clearance early during induction, as reflected by higher MRD-negative rates in CTC and ctDNA at IS. This early and profound tumor reduction is a prerequisite for sustained tumor control during maintenance, as demonstrated by our group in the MCL elderly trial [9]. We demonstrated that patients achieving MRD-negativity after induction have a stronger and sustained benefit from rituximab-maintenance resulting in prolonged remission.

In this series, 7/34 ibrutinib-treated patients relapsed after a median of 34 months, equivalent to end of maintenance, four of those had detectable MRD by qPCR during induction. The only patient who progressed and died early under ibrutinib had detectable CTC at IS. This patient harbored pathogenic mutations in *NRAS*, *CCND1*, and *ATM*. Activated MAPK pathway by *NRAS*^*Gln61Lys*^ could potentially rescue MCL cells from BTKi and increase MCL pathogenicity. Activating mutations in MAPK pathway-involved genes were detected in a fraction of ibrutinib-resistant CLL patients [37]. Despite being rare in MCL, further investigation of MAPK pathway and its contribution to ibrutinib resistance is warranted to resolve BTKi’s resistance mechanisms.

The direct comparison of ctDNA and CTC for MRD assessment showed higher sensitivity of ctDNA even when using IG-NGS with a sensitivity of 10^−6^ for CTC. However, this did not translate into more precise outcome prediction, as the best prognostication of the whole cohort was achieved by qPCR. When analyzing treatment arms separately, the significance of ctDNA compared to CTC remained ambiguous. Interestingly, ctDNA detection in arm A without ibrutinib was more predictive for adverse FFS and OS than CTC. This might reflect the early and profound CTC clearance by cytarabine-based immunochemotherapy leading to high MRD response rates of 86% in the PB as shown by our group before [38], while residual disease in LN is not properly eradicated by intensive immunochemotherapy alone.

In contrast, in ibrutinib-containing arms, only CTC detection by qPCR was predictive for adverse outcomes. This is somehow unexpected and cannot directly be explained by features of the different methods used to assess MRD. More likely, this observation reveals a different MCL cell dynamic under ibrutinib administration, MCL cells are mobilized from LN into the blood becoming detectable by MRD assessment. We assume that functionally, this “compartment shift” results in a maximal cytotoxic effect of immunochemotherapy, contributing to the high efficacy of the combination treatment. Furthermore, we speculate that ibrutinib/chemotherapy combination also leads to increased cytotoxicity in the LN compartment. This could be shown by a more detailed analysis of ctDNA-positive patients at IS, where ctDNA levels in patients receiving ibrutinib/chemotherapy were significantly higher than in patients receiving immunochemotherapy only. Assuming that most of the ctDNA reflects apoptotic MCL cells from the LN and BM, this finding indicates increased cell turnover induced by ibrutinib with subsequent more effective cell killing also in the LN. This could be due to ibrutinib’s disruption of BTK’s role in integrin signaling and CXCR4/CXCL12-mediated adhesion, reducing the cell’s ability to home to supportive niches and promoting cellular mobilization [39]. Our findings of tumor dynamics highlight the efficacy of ibrutinib in MCL treatment, specifically in combination with platin-based chemotherapy, and demonstrate the impact of ibrutinib early during induction treatment.

We demonstrated the feasibility of MRD assessment by capture-based sequencing of cfDNA, although it did not override CTC-based prognostication. The feasibility of serum cfDNA for MRD assessment in MCL was demonstrated earlier by Lakhotia et al. [18], however, ctDNA was assessed by tumor-specific IG clonotype detection, which in our hands is less sensitive than targeted sequencing. Similar to our results, ctDNA detection during induction treatment was associated with inferior outcomes, however, in that study, 34/40 (85%) ctDNA-negative patients at EoI progressed after a median of 22.8 months, suggesting a limited sensitivity and negative-predictive-value of this approach. To draw broader conclusions on the preferred MRD approach for prognosis assessment in MCL, larger patient cohorts and different treatment timepoints should be comparably investigated.

Taken together, we hypothesize a scientific rationale for ibrutinib’s mechanism of action by stimulating MCL cell mobilization from the LN into the periphery, hence improving the efficiency of immunochemotherapy as demonstrated in CLL by Chen et al. [40].

Besides direct anti-tumor activity, long-term disease control in MCL by ibrutinib could also be mediated by effects on T-cell compartment remodeling, enhancing T-cell responses against MCL cells, and supporting tumor-immune control. This has been shown in CLL where among others, Davis et al. observed a T-cells increase after long-term BTKi exposure in vitro [41].

Using Flow-cytometry immunomonitoring in eight patients with/without ibrutinib, we observed T-cell kinetic changes in T-cell immune profiles represented by a decline of inhibitory T-cell phenotypes in ibrutinib-treated patients, suggesting an improved immune control in this limited group. Concordant results for CD4+-T-cell populations were reported by Niemann et al. for CLL patients receiving ibrutinib [42]. Our results are preliminary and not statistically validated due to the small size, however warrant further investigation and worth consideration for further experimental designs.

Overall, we demonstrate ibrutinib’s role in early MRD eradication and long-term disease control and highlight liquid biopsy’s potential for real-time molecular profiling in MCL.

## Supplementary information

Supplemental Figures

Supplemental Tables

## Data Availability

Raw and processed sequencing data are available from the corresponding author upon reasonable request to: c.pott@med2.uni-kiel.de.
