# Supplementary material for: Noninvasive genotyping and early disease dynamics demonstrate the efficacy of ibrutinib in combination with immunochemotherapy in patients with mantle cell lymphoma treated in the TRIANGLE trial
Source: Leukemia. 2025 Nov 3;40(1):95–105. doi: 10.1038/s41375-025-02787-0 (PMC12789019; doi:10.1038/s41375-025-02787-0)
Supplement: Supplementary file 1 — Supplemental Figures [file 41375_2025_2787_MOESM1_ESM.docx]

**Supplementary Material**

**Noninvasive genotyping and early disease dynamics demonstrates the efficacy of ibrutinib in combination with immunochemotherapy in patients with mantle cell lymphoma treated in the TRIANGLE trial**

Mouhamad Khouja^1^, Elisa Genuardi^2^, Simone Ferrero^2^, Anna Laqua^1^, Beatrice Alessandria^2^, Onno J.H.M. Verhagen^3^, Christa H.E. Homburg^3^, Ramón García Sanz^4^, Alejandro Medina Herrera^4^, Vincent H.J. van der Velden^5^, Maria Gomes da Silva^6^, Paula Gameiro^6^, Jeanette Doorduijn7, Eva Giné^8^, Carlo Visco^9^, Monika Brüggemann^1,10^, Claudia D Baldus^1, 10^, Marco Ladetto^11^, Christian Schmidt^12^, Martin Dreyling^12^, Linmiao Jiang^13^, Eva Hoster^13^, Nikos Darzentas^1^, Karol Pal^14^, Guranda Chitadze^1,10^, James Peter Stewart^15^, David Gonzalez^15^, Christiane Pott^1^ on behalf of the European MCL network

Affiliations:

1 Second Medical Department, University Hospital Schleswig-Holstein, Kiel, Germany.

2 Department of Molecular Biotechnologies and Health Sciences - Hematology Division, Università di Torino, Torino, Italy.

3 Department of Immunocytology, Sanquin Diagnostic Services, Amsterdam, The Netherlands.

4 CIBERONC, Hospital Universitario de Salamanca-IBSAL, Universidad de Salamanca, Salamanca, Spain.

5 Laboratory Medical Immunology, Department of Immunology, Erasmus MC, University Medical Center Rotterdam, Rotterdam, The Netherlands.

6 Haematology Unit, Instituto Português de Oncologia de Lisboa Francisco Gentil, 1099-023 Lisbon, Portugal.

7 Department of Hematology, Erasmus MC Cancer Institute, University Medical Center Rotterdam, Rotterdam, Netherlands

8 Hematology Department, Hospital Clínic de Barcelona, IDIBAPS, Barcelona, Spain.

9 Department of Engineering for Innovative Medicine, University of Verona, Verona, Italy

10 Clinical Research Unit CATCH ALL, Christian-Albrechts-University of Kiel, Kiel, Germany.

11 SC Ematologia Azienda Ospedaliera Santi Antonio e Biagio e Cesare Arrigo, Alessandria, Italy.

12 Department of Medicine III, University Hospital, Ludwig-Maximilian-University, Munich, Germany.

13 Institute for Medical Information Processing, Biometry, and Epidemiology (IBE), LMU University Munich, Munich, Germany.

14 CEITEC MU - Central European Institute of Technology, Masaryk University, Brno, Czech Republic.

15 Precision Medicine Centre, Patrick G Johnston Centre for Cancer Research, Queen’s University Belfast, Belfast, United Kingdom.

Corresponding author

Prof. Dr. med. Christiane Pott

Second Medical Department

University Hospital Schleswig-Holstein, Campus Kiel

Arnold-Heller Str. 3

24105 Kiel

Germany

Phone: ++49-431-500-24981

Fax: ++49-431-500-22554

Email: [c.pott@med2.uni-kiel.de](mailto:c.pott@med2.uni-kiel.de)

Figure S1 Failure-free (FFS) and Overall survival (OS) for patients (A) with (red line) and (B) without (blue line) cfDNA analysis in the whole TRIANGLE cohort, (C-D) FFS and OS survival for patients analyzed in this study depicted by treatment arm. Patients treated in arms A+I/I are represented by a red line while patients treated in arm A are represented by a black line.


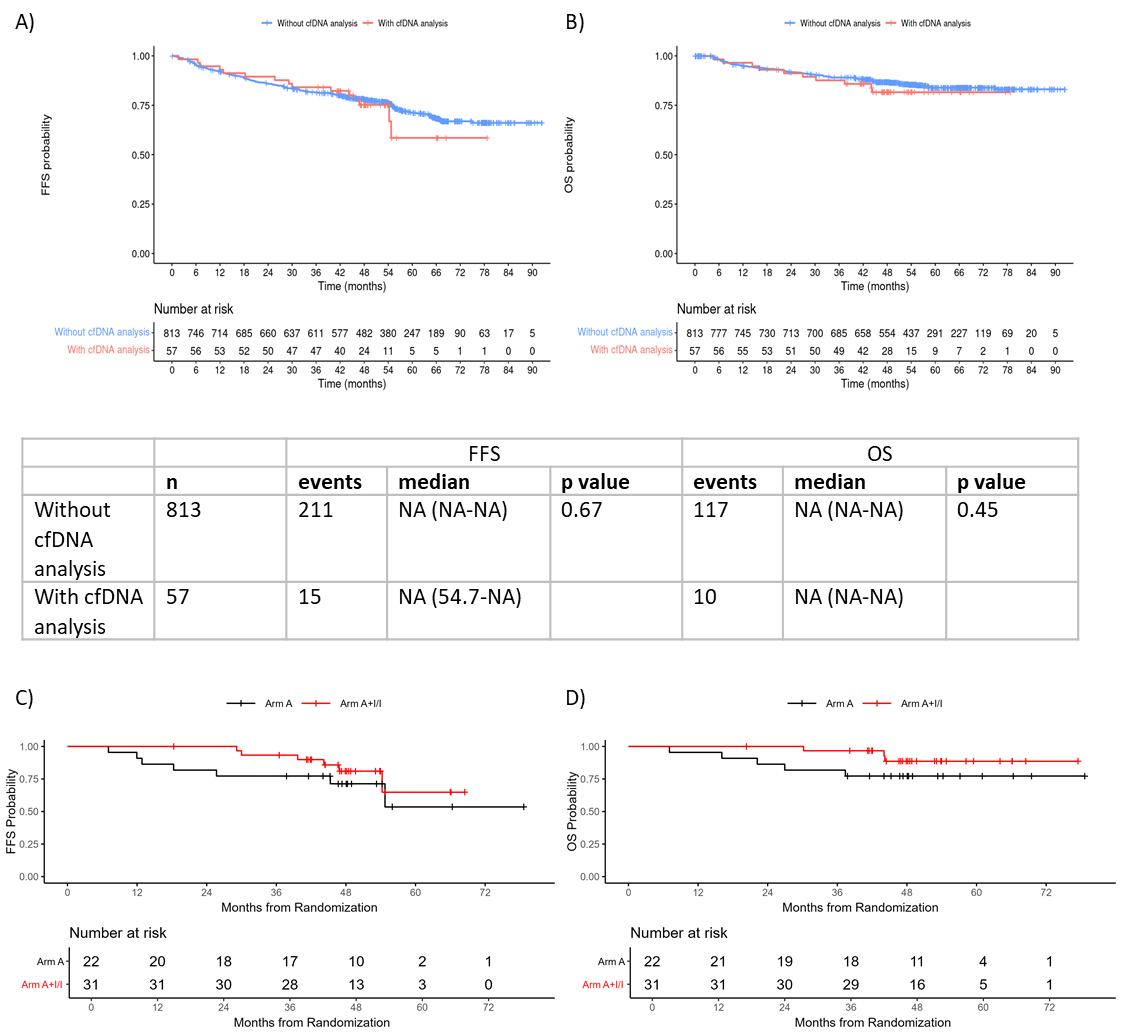


Figure S2 Molecular profiles of six patients with somatic variants identified in cfDNA only but not gDNA isolated from PB cells.


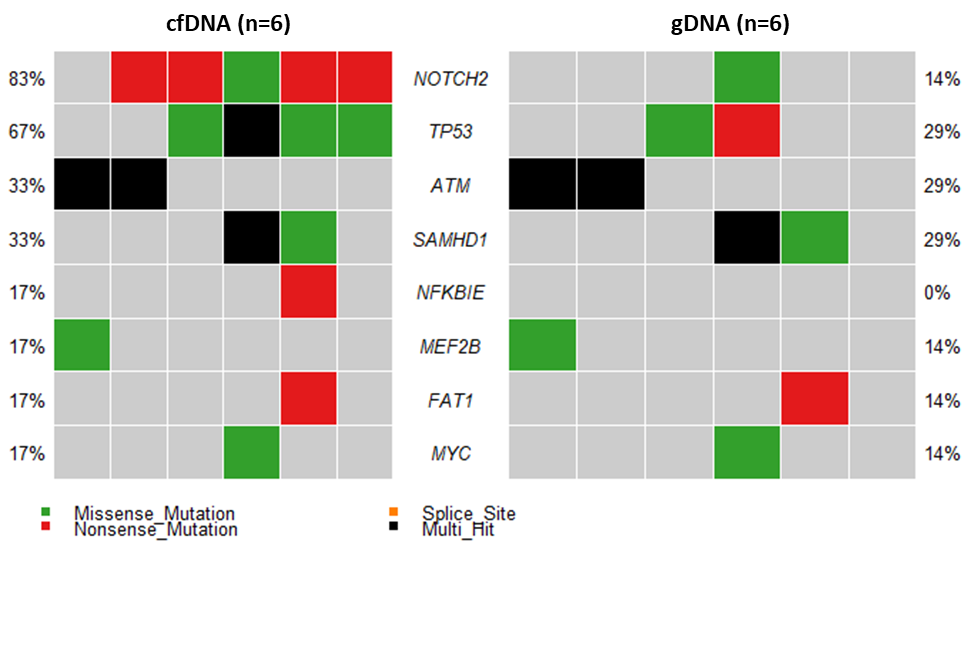


Figure S3
